# Stress Relaxation and Grain Growth Behaviors of (111)-Preferred Nanotwinned Copper during Annealing

**DOI:** 10.3390/nano13040709

**Published:** 2023-02-13

**Authors:** Jyun-Yu Lai, Dinh-Phuc Tran, Shih-Chi Yang, I-Hsin Tseng, Kai-Cheng Shie, Jihperng Leu, Chih Chen

**Affiliations:** 1 Department of Materials Science and Engineering, National Yang Ming Chiao Tung University, Hsinchu 30010, Taiwan; 2 Department of Materials Science and Engineering, National Chiao Tung University, Hsinchu 30010, Taiwan

**Keywords:** nanotwinned copper, thermal stress, stress relaxation, microstructure, grain growth

## Abstract

Highly (111)-oriented nanotwinned Cu (nt-Cu) films were fabricated on silicon wafers for thermal-stress characterization. We tailored the microstructural features (grain scale and orientation) of the films by tuning the electroplating parameters. The films were heat-treated and the relaxation behaviors of thermal stresses in the films were explored using a bending beam system. Focused ion beam (FIB) and electron back-scattered diffraction (EBSD) were then employed to characterize the transformations of the microstructure, grain size, and orientation degree of the films. The results indicated that the degree of (111)-preferred orientation and grain size significantly decrease with increasing the current density. The nt-Cu films with a higher degree of (111)-preferred orientation and larger grains exhibit the slower rates of stress relaxation. The film with larger grains possesses a smaller grain boundary area; thus, the grain boundary diffusion for the thermal-stress release is suppressed. In addition, the induced tensile stress in the films with larger grains is smaller leading to the difference in microstructural changes under annealing.

## 1. Introduction

Currently, the collision of the COVID-19 pandemic effect and fast-surging demand has intensified the already severe global shortage for computing chips used in every corner, from consumer mobile phones to electric vehicles (EVs). In the semiconductor industry, copper (Cu) is considered as the most commonly used interconnect for fan-out wafer level packaging thanks to its superior electrical and thermal conductivity [1,2,3,4,5,6]. In order to enhance the computing performance of electronic devices, logic chips and interconnects are required to scale down significantly [7,8,9]. During operation, Cu lines are subjected to a high current density resulting in severe failures related to electromigration (EM), stress migration, and Joule heating [10,11,12]. Thus, studies on these reliability issues are needed.

The mechanical strength of regular Cu is not satisfactory for high temperature and high-mechanical-required applications. Grain refinement is a well-known method to enhance the mechanical strength of pure copper. However, it normally accompanies ductility loss. Incorporating nanotwins in Cu can be considered as the most effective approach to suppress EM failures [13,14,15,16] and to enhance its mechanical properties [17,18,19,20,21,22,23,24,25] while its electrical resistance can remained approximately unchanged. In recent years, nano-twinned copper (nt-Cu) has drawn significant attention due to its high mechanical strength and conductivity, low manufacturing cost, and oxidation [26,27,28]. Thus, it can be ideally used as interconnects in advanced packaging technology. Nt-Cu can be fabricated by magnetron sputtering, plastic deformation, chemical vapor deposition, 3D printing, and electroplating. The enhancement in mechanical strength originates from the impediment of the twin boundaries to dislocation movement. The strength of nt-Cu can be enhanced by reducing its twin thickness. However, refining its twin thickness below a critical value will lead to a severe decline in mechanical strength [26,27]. This phenomenon is defined as a Hall–Petch softening effect [28]. Other common methods to enhance the mechanical properties of copper are solution and aging precipitation strengthening. However, they will cause a remarkable loss of electrical conductivity [29] and thus are not suitable for applications requiring materials with high conductivities.

Nanotwinned materials could be employed in various applications such as energy conversion [30,31,32,33], an electrocatalyst for CO_2_ reduction [34], a photochemical system [35,36,37], thermal management [38], and biological sensing [39,40,41]. In the semiconductor industry, the mismatch of the coefficient of thermal expansion (CTE) between Cu lines and dielectric materials is of critical concern. Thermal stress may be generated leading to the severe warpage and failure of wafer [42,43,44]. Under a high temperature, severe compressive and/or tensile stresses are induced in Cu lines. The self-diffusion of Cu atoms can form hillocks on the Cu surfaces as subjected to EM stressing [45,46,47,48]. Previous studies have shown that voids are likely to form as tensile stress is significant [47,49,50]. They may alter the distribution of current density and facilitate the Cu depleting process [51]. Those defects can accumulate in the Cu lines and eventually result in the complete failures of electronic devices.

Since the stress relaxation of the Cu films as a function of thermal history is directly related to the diffusion of Cu atoms, in this study we electroplated highly (111)-oriented nt-Cu films on silicon wafers for thermal-stress characterization. We tailored the microstructures of the nt-Cu films by tuning the electroplating parameters. The nt-Cu films were further heat-treated, and their thermal stability was characterized. The relationship between thermal-stress behaviors and microstructures was then explored using a homemade bending beam system. This study provides insights on the thermal-stress behaviors of thin nt-Cu films under thermal loading.

## 2. Materials and Methods

In this study, various Cu films were deposited on double-side polished silicon (Si) wafers (1 cm × 5 cm) with a thickness of 760 µm for stress measurements. The Si wafer consisted of 100-nm SiO_2_, 100-nm TiW adhesion, and 200-nm Cu seed layers. The seed films were deposited by sputtering (Oerlikon Cluster Line 300, Pfäffikon, Switzerland). In order to remove the organic contaminants on the surface, the substrates were cleaned with acetone and isopropyl. They were subsequently soaked in a solution of citric acid and DI water for a few seconds to remove oxides from the surfaces prior to electroplating. The electrolyte contained 0.8 M of high-purity CuSO_4_ solution, 40 ppm of Cl^−^, and 20 mL/L of a commercial additive (DP101, Chemleader, Inc., Hsinchu, Taiwan). The HCl solution was employed to increase the deposition rate and improve the crystallinity of the Cu films while the additive was for the formation of the (111)-oriented nt-Cu. The electrodepositions were conducted at 25 and 35 °C with a stirring rate of 1200 r.p.m. Various electroplating current densities of 4, 8, and 12 ASD (A/dm^2^) were tuned. The different current densities of 4, 8, and 12 ASD were utilized in the current study because such electroplating parameters are suitable for laboratory-scale investigation. The microstructural features (grain scale and orientation) of the nt-Cu films could be altered by tuning the electroplating parameters. The thermal-stress behaviors of the films were then correlated with their microstructural changes. The Cu films were deposited with a thickness of 3.8 μm.

**Figure 1:**
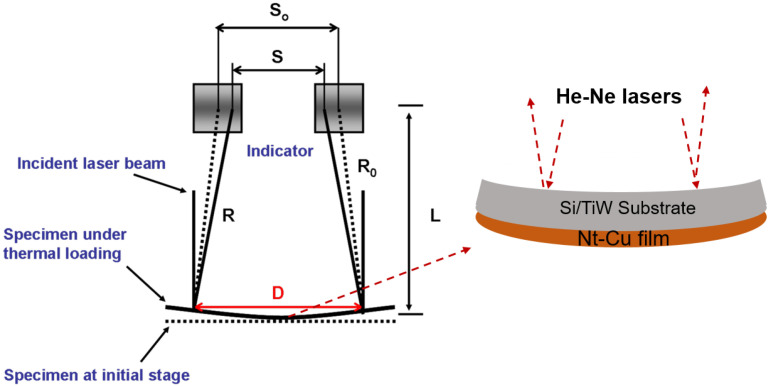
Schematic of the thermal-stress measurement. The dotted and solid lines represent the films before and during heating, respectively.

During the annealing process, thermal stress was determined by measuring the curvature change of the films. A sampling rate of 0.05 Hz was set to record the curvature changes. It was due to the CTE mismatch of the silicon substrate and Cu film. The thermal-stress measurements were entirely carried out in a home-built bending beam system, as shown in Figure 1. The films were heated to 200 °C in a vacuum (8 × 10^−3^ torr) at a heating rate of 5 °C/min. In the semiconductor industry, a processing temperature below 200 °C is favorable and highly recommended for chip packaging. A higher annealing temperature may be detrimental to other electronic components. In fact, we aimed to imitate the actual processes in advanced packaging and to address the stress relaxation and grain growth behaviors of such Cu films during thermal processing. The thermal behaviors of the films under different annealing temperatures could be an interesting topic for further investigation. The temperature was then held for 10 h and naturally cooled down to room temperature. The average in-plane stress in the films was determined using Stoney’s Equation [52,53,54],

(1) $$\sigma =\frac{E_{s}t_{s}^{2}}{6\left(1-\nu_{s}\right)t_{f}}\left(\frac{1}{R}-\frac{1}{R_{0}}\right)$$

where *σ* is the thermal stress in the film, *E*_s_ and *ν*_s_ are the Young’s modulus and Poisson’s ratio of the substrate, *t*_s_ and *t*_f_ are the thickness of the substrate and the film, *R* and *R*_0_ are the curvature radius of the film/substrate and the bare substrate, respectively. By this manner, we could obtain the variation of thermal stress in the Cu films as a function of temperature. In this study, we neglected the effect of the adhesion TiW layer on the stress behaviors during annealing because its thickness was much smaller than that of the electroplated nt-Cu films. For the microstructural analysis, focused ion beam (FIB, FEI Nova 2000, Hillsboro, OR, USA) and electron back-scattered diffraction (EBSD, JEOL JSM-7800F, Tokyo, Japan) were employed to observe the microstructure, grain size, and orientation of the films. The grain size and orientation of the nt-Cu films deposited at different temperatures were also analyzed using EBSD and FIB images. In order to perform the stress analysis, a diced sample with a physical dimension of 1 cm × 5 cm was attached on a holder of the bending beam system in a vacuum (8 × 10^−3^ torr). Under the annealing process, the nt-Cu films were subjected to a warpage effect leading to changes in Cu film curvature. A helium–neon (He-Ne) laser source with a wavelength of 632.8 nm was used. The laser source was divided into two laser beams by a beam splitter, and they were reflected by two mirrors. The distance between the two light sensors and the films was set as 45 mm. The polished silicon substrate sides of the samples were set upward to reflect the laser beam, as shown in Figure 1. During annealing, the curvature changes were determined by the reflection of the parallel laser beams detected by 2D position sensors. The thermal-stress relaxation was then calculated and characterized by recording the changes of film curvatures (Equation (1)). Additionally, the stress relaxation behaviors of the nt-Cu films with different grain sizes and ratios of (111) orientation under the annealing were analyzed.

## 3. Results and Discussion

**Figure 2:**
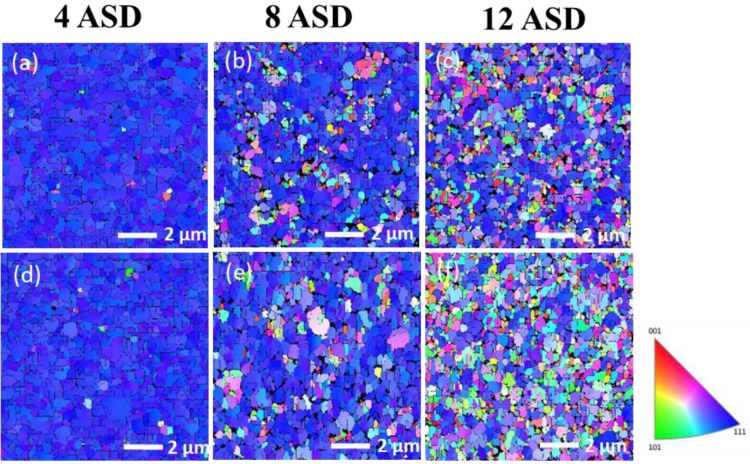
Top-view EBSD images of the as-fabricated (**a**–**c**) and annealed (**d**–**f**) nt-Cu films deposited with various current densities of 4, 8, and 12 ASD, respectively. The nt-Cu films were electrodeposited at 25 °C and the annealing was conducted at 200 °C for 10 h.

**Figure 3:**
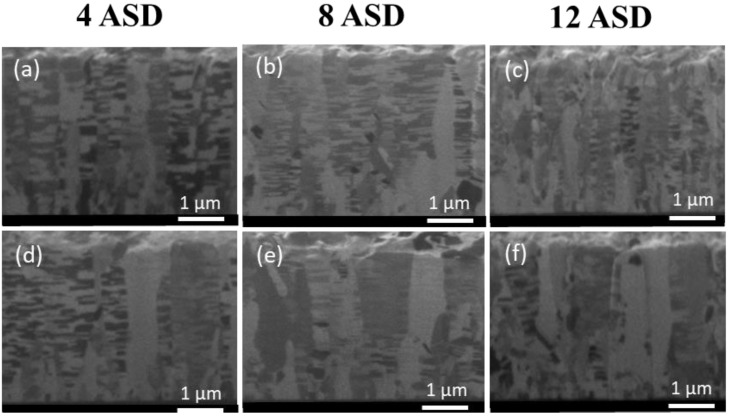
Cross-sectional FIB images of the as-fabricated (**a**–**c**) and annealed (**d**–**f**) nt-Cu films deposited with various current densities of 4, 8, and 12 ASD, respectively. The nt-Cu films were electrodeposited at 25 °C and the annealing was conducted at 200 °C for 10 h.

In this study, Cu films with different orientation ratios were fabricated by adjusting the electroplating current density. The microstructures of the Cu films were then analyzed by FIB and EBSD. The top-view EBSD images of the as-electroplated and annealed Cu films deposited under various current densities are shown in Figure 2. The blue regions in the EBSD orientation map represent the (111) grains in the nt-Cu films. The orientation ratio and grain size are listed in Table 1 and Table 2. It was found that the ratio of (111)-preferred orientation and grain size significantly decreased with increasing the current density (Figure 2a–c). After annealing at 200 °C for 10 h, the Cu grain size slightly increased. No obvious change in the grain orientation was found when electroplated with a low current density (4 or 8 ASD). This indicates a great thermal stability of such nt-Cu films because their coherent twin boundaries are strongly stable at elevated temperatures [55,56,57]. In addition, twins of Cu can alter their surface structure and grain boundary leading to the stability of the twin boundaries and surface [58,59]. As electroplated under 12 ASD, the degree of (111) orientation apparently decreased after the annealing. Figure 3 shows the cross-sectional FIB images of the nt-Cu films deposited with different current densities with and without annealing. It can be observed that the columnar structure of the films was less obvious as electroplated with a high current density. Twin spacing and grain size decreased with increasing the current density (Figure 3a–c). They further increased to some extents after annealing at 200 °C for 10 h (Figure 3d–f). These results are consistent with the aforementioned EBSD analysis.

In the film, the total strain includes elastic and plastic strains. During isothermal process, the total strain of the film can be considered as a constant. In other words, the plastic strain of a certain material increases while the elastic strain decreases. The stress relaxation rate can be expressed as follows,

(2) $$\dot{\sigma }=-M(T){\dot{\varepsilon }}_{p}$$

where ${\dot{\varepsilon }}_{p}$ and *M* are the inelastic strain rate and biaxial modulus (as a function of temperature, *T*). The biaxial modulus can be determined by identifying the slope of temperature changes. The strain rate of the film can be expressed by a linear creep relation as follows [60],

(3) $${\dot{\varepsilon }}_{p}=\frac{\sigma }{\eta }$$

where *η* is the creep viscosity which is dependent on temperature. Substituting Equation (3) into Equation (2), we can express the stress in the film as a function of time,

(4) $$\sigma (t)=\sigma_{0}\exp(-t.\frac{M}{\eta })$$

However, to fully describe the stress relaxation in the film, an amount of residual stress after an infinite time of thermal loading should be included. Thus, Equation (4) can be expressed as,

(5) $$\sigma (t)=\sigma_{\infty }+\Delta \sigma_{1}\exp(-\frac{t}{\tau_{1}})+\Delta \sigma_{2}\exp(-\frac{t}{\tau_{2}})$$

where $\tau_{1}=\frac{\eta }{M}$ is the relaxation time constant, and α_∞_ is the residual stress at infinite time.

**Figure 4:**
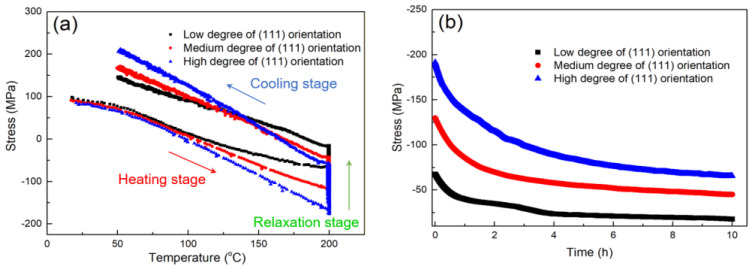

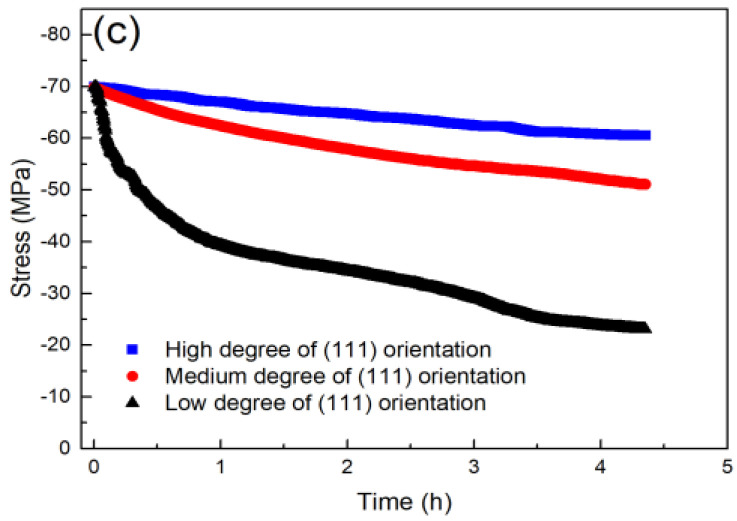
(**a**) Thermal-stress behaviors of the nt-Cu films with different degrees of (111) orientation. Note that the films with high, medium, and low degrees of (111) orientation, respectively, represent the samples shown in Figure 2a–c. (**b**) Stress relaxation of the films with different degrees of (111) orientation. (**c**) Stress relaxation plotted from the same value of initial compressive stress at 200 °C.

In this study, the thermal-stress behaviors of the nt-Cu films were characterized using a bending beam system in a vacuum. They were heated at a heating rate of 5 °C/min to 200 °C and the temperature was then maintained for 10 h to study the stress relaxation of the films. Finally, the films were slowly cooled down to room temperature. During such annealing, the induced thermal stress of the films was recorded by measuring the curvatures of the films. Figure 4a shows the thermal-stress behaviors of the nt-Cu films with different degrees of (111) orientation. As the temperature increased, the tensile stress converted to compressive stress because of the CTE mismatch. The compressive stress reached a maximum value at the beginning of the annealing (200 °C). During the annealing, it gradually relaxed to nearly zero. As the sample cooled down, it transformed to a greater tensile stress compared to its initial state. During the temperature changes, the stress relaxation was dominated by the interaction between the plastic flow and grain boundary diffusion [61]. It resulted from the minimization processes of the defect/twin/strain and interface energies [62]. We found that, under the annealing process, the nt-Cu films with a higher degree of (111) orientation exhibited a greater range of thermal stress. Their nanotwin structures with superior mechanical properties could withstand higher compressive and tensile stresses and store a larger amount of strain energy. Under a high annealing temperature, the nanotwins could stabilize the Cu microstructure and store more thermal stress. Thus, grain growth could only occur at high temperatures. The relaxation behavior of thermal stress versus time is shown in Figure 4b. It was found that the nt-Cu films with higher degrees of (111)-preferred orientation reached its stable state slower than the lower ones. In order to avoid the difference in the deformation mechanism in relaxation with different stress levels, we compared the relaxation from the same stress level. As shown in Figure 4c, the nt-Cu films with highly (111)-preferred orientation apparently exhibited less stress relaxation.

**Figure 5:**
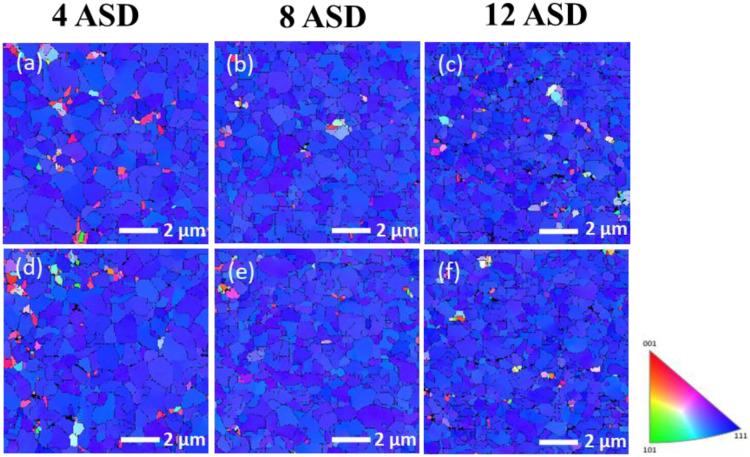
Top-view EBSD images of the as-fabricated (**a**–**c**) and annealed (**d**–**f**) nt-Cu films deposited at 35 °C with various current densities of 4, 8, and 12 ASD, respectively. The annealing was conducted at 200 °C for 10 h.

**Figure 6:**
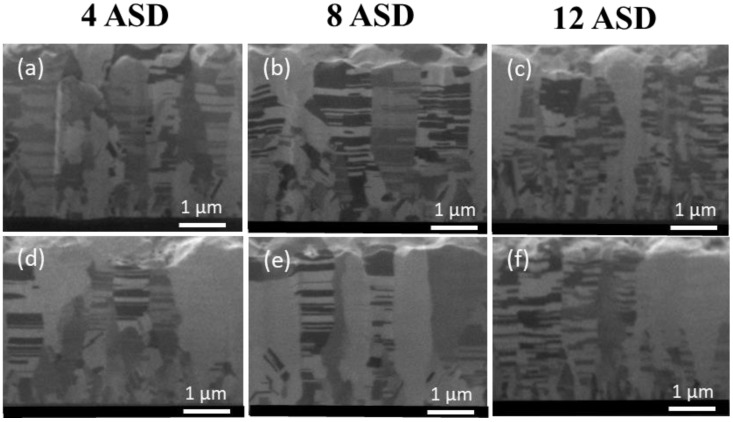
Cross-sectional FIB images of the as-fabricated (**a**–**c**) and annealed (**d**–**f**) nt-Cu films deposited with various current densities of 4, 8, and 12 ASD, respectively. The nt-Cu films were electrodeposited at 35 °C and the annealing was conducted at 200 °C for 10 h.

In this study, we also conducted the electrodeposition of the nt-Cu films at 35 °C to tailor their microstructure features. The top-view EBSD images of the nt-Cu films deposited at different current densities are shown in Figure 5a–c. All samples possessed high degrees of (111)-preferred orientations. The effect of current density on the orientation was not obvious but only significant on the grain size of the Cu films. The grain sizes deposited at 4, 8, and 12 ASD were estimated as 0.64, 0.48, and 0.39 µm, respectively. Figure 5d–f shows the typical EBSD images of the nt-Cu films after annealing at 200 °C for 10 h. The variations in grain orientation degree and size of the as-deposited and annealed nt-Cu films are summarized in Table 3 and Table 4. No obvious variations on grain orientation were found indicating the orientation stability of the films. However, their grains apparently grew after the annealing. The profound driving force of such nt-Cu films may assist anisotropic grain growth and eliminate grain boundaries under the annealing condition. Figure 6 shows the cross-sectional FIB images of the highly (111)-oriented nt-Cu films with different grain sizes before and after annealing at 200 °C for 10 h. It can be observed that their columnar structures remained unchanged after the annealing. The grain size of the films increased with decreasing the electroplating current density. It further increased under the annealing condition.

As shown in Table 1 and Table 3, the grain size and (111) ratio of the nt-Cu films decreased with increasing the electroplating current density. We also found that the degree of (111) orientation of the films deposited at 25 °C was smaller than at 35 °C. These results can be elucidated as follows. First, at the beginning stage of electrodeposition, a high current density causes an increase in the number of Cu nuclei. The closer distance and greater number of the densely formed Cu nuclei on the substrate result in a larger number of growing grains and lead to a decrease in grain size. The high current density squeezes the adjacent growing grains. Such grains push and overlap other grains to form a transition layer (nanoscale grain region). It has been reported that the thickness of transition layers increases with increasing anodic current density [63]. The growth of columnar grains is strongly correlated with the uniformity of transition layers. The columnar grains will be slanted and less perpendicular to the substrate as continuous depositing on a thicker transition layer (less uniform). Note that the twin planes of face-centered-cubic (FCC) Cu are (111). A lower perpendicularity of columnar grains leads to a lower degree of (111) orientation. Thus, under a high current density, a low percentage of (111)-oriented grains was obtained. 

Second, at a higher electrolyte temperature, the nuclei population tends to decrease rather than increase in the chloride solution. It can be attributed to the desorption of chloride ions, facilitating the growth of the already formed nuclei instead of forming new ones [64]. Thus, the thickness of transition layers and slant degree of columnar nt-Cu grains could decrease as electroplated at a high temperature. The transition layer was uniform. As a result, the (111) ratio of the films electroplated at 35 °C was greater than at 25 °C. In other words, the nt-Cu films deposited at a low electrolyte temperature exhibited poor crystallinity and low adherence to the substrate. In addition, at a high temperature, the surface diffusion rate of Cu atoms is large. Under the electric bias, the newborn Cu atoms can easily diffuse along the substrate and aggregate to form Cu nanocrystals [65]. However, at a low temperature, the reaction rate decreases, and the deposition process is dominated by the reaction rate rather than the diffusion rate of Cu ions in the solution. The very-first-born Cu atoms do not diffuse fast enough. They cannot find their lowest surface energy sites and thus randomly distribute at the reaction locations leading to the poor crystallinity of the films.

**Figure 7:**
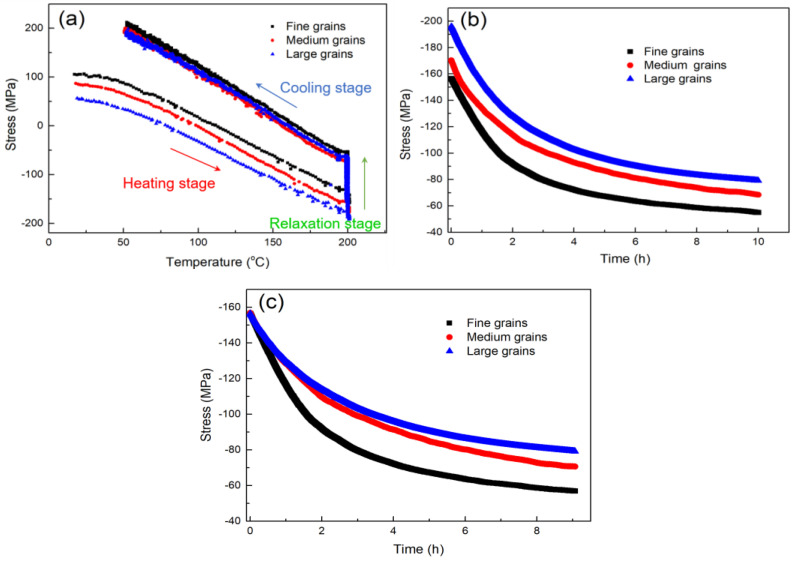
(**a**) Thermal-stress behaviors of the nt-Cu films with different degrees of (111) orientation. (**b**) Stress relaxation of the films with different degrees of (111) orientation. (**c**) Stress relaxation plotted from the same value of initial compressive stress at 200 °C. The nt-Cu films were electrodeposited at 35 °C.

We also characterized the thermal-stress behaviors of these aforementioned nt-Cu films using the homemade bending beam system. Figure 7a shows the thermal-stress behaviors of the nt-Cu films with different grain sizes and degrees of (111) orientation. We found that all the films responded in the same manner to the thermal loading. The rates of thermal-stress changes were comparable. However, due to the difference of the initial tensile stress, the stress magnitudes of each nt-Cu film during the annealing were varied (Figure 7a). This caused the larger compressive stress at 200 °C. Figure 7b shows that the nt-Cu films with different grain sizes exhibited a similar rate of stress relaxation, and the time to reach their steady states was comparable. However, under a similar stress gradient, the thermal stress in the nt-Cu films with larger grains decreases slower compared with the smaller grain ones. This could be attributed to two possible reasons. First, the nt-Cu film with larger grains possesses a smaller area of grain boundaries. Grain boundary diffusion for the release of thermal stress is thus suppressed. Second, the microstructural changes of the films with different grain sizes under annealing are different. Their grain boundaries rearrange and densify under annealing, leading to the expansion in volumes. Tensile stresses may be induced in the films, which can be calculated by the following equation [4]:

(6) $$\sigma =\sigma_{xx}=\sigma_{yy}=\frac{E}{{\left(1-v\right)}_{film}}\Delta a\left(\frac{1}{L_{o}}-\frac{1}{L}\right)$$

where *E* and *v* are the Young’s modulus and Poisson’s ratio of the film, respectively; ∆*a* is the extra grain boundary volume per unit area after annealing; *L*_o_ and *L* are the grain diameters before and after annealing. Assuming that, ∆*a* = 1 Å, the stresses induced by the grain boundary densification are summarized in Table 5. Obviously, the induced tensile stress in the films with larger grains was much smaller compared to that with the smaller ones. 

Currently, Cu-Cu bonding is of wide interest in the semiconductor packaging industry. It has been reported that highly (111) nt-Cu possesses the highest surface diffusivity and lowest oxidation compared to regular Cu [66]. The bonding temperature can be significantly lowered using such nt-Cu. It can be further reduced with the optimal grain size and (111) orientation ratio of the nt-Cu films by tuning the electroplating current density. In addition, practical solutions to remove completely interfacial voids in Cu-Cu bonding are still unknown, leaving many related questions unanswered. Thus, more considerations are needed. In general, such unique Cu is not only applicable for microelectronics but also in various applications from bio sensing, aerospace, photo catalytic, critical safety to mechanical bearing structural industries. Future research on the nt-Cu films is not limited to any particular field but their combinations.

## 4. Conclusions

In summary, we deposited highly (111)-oriented nt-Cu on silicon wafers and tailored the microstructures of the nt-Cu films by tuning the electroplating parameters (temperature and current density). The films were further heat-treated to some extents. The relationship between the stress relaxation behaviors and microstructures was characterized using a bending beam system. The transformations of the microstructure, grain size, and orientation degree of the films were then explored by FIB and EBSD. As electroplated at 25 °C, the percentage of (111) orientation and grain size of the nt-Cu films markedly decreased with an increase in electroplating current density. However, the effect of the current density on the nt-Cu microstructures was not obvious as electroplating the films at a high temperature (35 °C). We also found that, as the films were annealed at 200 °C for 10 h, their grain sizes slightly increased. The grain orientation of the films deposited under a low current density (4 or 8 ASD) did not obviously change after annealing. This result indicates a good thermal stability of such nt-Cu films thanks to the great stability of their coherent twin boundaries. It was also found that the (111) ratio of the films electroplated at a low temperature (25 °C) was smaller than that deposited at a high one (35 °C). This could be attributed to the increase in slant degree of the columnar nt-Cu grains and the decrease in the reaction rate. We found that the Cu films with higher (111)-preferred orientation and larger grains showed slower stress relaxations. The films with larger grains have a smaller area of grain boundaries. Thus, the grain boundary diffusion for the release of thermal stress was suppressed. In addition, under annealing, the induced tensile stress in the films with larger grains was much smaller compared to that with the smaller ones leading to the difference in the microstructural changes.

## Figures and Tables

**Table 1:** Degree of (111) orientation and grain size of the films deposited at 25 °C.

Samples (As-Fabricated)	(111) Ratio (%)	Average Grain Size (µm)
25 °C, 4 ASD	98.5	0.60 ± 0.29
25 °C, 8 ASD	73.4	0.41 ± 0.19
25 °C, 12 ASD	43.5	0.33 ± 0.14

**Table 2:** Degree of (111) orientation and grain size of the nt-Cu films deposited at 25 °C and annealed at 200 °C for 10 h.

Samples (After Annealing)	(111) Ratio (%)	Average Grain Size (µm)
25 °C, 4 ASD	98.1	0.64 ± 0.25
25 °C, 8 ASD	69.7	0.48 ± 0.21
25 °C, 12 ASD	35.9	0.36 ± 0.15

**Table 3:** Degree of (111) orientation and grain size of the films deposited at 35 °C.

Samples (As-Fabricated)	(111) Ratio (%)	Average Grain Size (µm)
35 °C, 4 ASD	95.2	0.64 ± 0.38
35 °C, 8 ASD	97.8	0.48 ± 0.33
35 °C, 12 ASD	95.9	0.39 ± 0.29

**Table 4:** Degree of (111) orientation and grain size of the nt-Cu films deposited at 35 °C and annealed at 200 °C for 10 h.

Samples (After Annealing)	(111) Ratio (%)	Average Grain Size (µm)
35 °C, 4 ASD	94.3	0.94 ± 0.37
35 °C, 8 ASD	97.6	0.79 ± 0.33
35 °C, 12 ASD	97.3	0.59 ± 0.32

**Table 5:** Influence of grain size on the amount of stress relaxation.

Samples	As-Fabricated, Grain Size (µm)	After Annealing, Grain Size (µm)	Stress (MPa)
35 °C, 4 ASD	0.64 ± 0.38	0.94 ± 0.37	11.7
35 °C, 8 ASD	0.48 ± 0.33	0.79 ± 0.33	19.2
35 °C, 12 ASD	0.39 ± 0.29	0.59 ± 0.32	20.4

## Data Availability

The raw/processed data required to reproduce these findings cannot be shared at this time due to legal or ethical reasons.
